# Chemotherapy-Induced Alopecia in Breast Cancer Patients: Treatment-Specific Incidence and Risk of Persistent Hair Loss

**DOI:** 10.3390/cancers18050861

**Published:** 2026-03-07

**Authors:** Simonetta I. Gaumond, Sophie Shrestha, Isabella Kamholtz, Gabriela E. Beraja, Joaquin J. Jimenez

**Affiliations:** 1 Department of Biochemistry and Molecular Biology, University of Miami Miller School of Medicine, Miami, FL 33136, USA; sxg1204@miami.edu; 2 Dr. Phillip Frost Department of Dermatology and Cutaneous Surgery, University of Miami Miller School of Medicine, Miami, FL 33136, USA

**Keywords:** chemotherapy-induced alopecia (CIA), chemotherapy, breast cancer, alopecia, hair loss, adverse event, side effect, toxicity, taxanes, anthracyclines, cyclophosphamide

## Abstract

Chemotherapy-induced alopecia (CIA) is one of the most common and visible toxicities of breast cancer treatment, yet its true incidence, severity, and long-term outcomes remain inconsistently reported. Although CIA is frequently cited as affecting approximately 65% of patients and persistent alopecia has historically been considered uncommon (1–15%), emerging data suggest a substantially greater burden. We conducted a scoping review of PubMed, EMBASE, SCOPUS, and Cochrane databases to synthesize regimen-specific evidence on the incidence, severity, and persistence of CIA in breast cancer patients. Anthracycline- and taxane-based regimens were associated with the highest risk, with severe alopecia reported in more than 70% of patients and rates approaching 90–100% in combination regimens. Cyclophosphamide further amplified acute CIA when combined with doxorubicin, with reported incidence up to 93%. In contrast, capecitabine and vinorelbine were consistently associated with lower alopecia incidence. Importantly, CIA was not uniformly reversible. Persistent CIA (pCIA) occurred in up to 67% of patients treated with doxorubicin-based regimens and nearly 50% of those receiving docetaxel combinations, substantially higher than historically reported. Despite its high frequency and potential permanence, CIA remains underreported in oncology trials and insufficiently addressed in survivorship care. Recognizing CIA as both an acute toxicity and a potential long-term survivorship concern underscores the need for standardized reporting, longitudinal follow-up, and development of effective preventive strategies in breast cancer care.

## Simple Summary

Chemotherapy-induced alopecia is one of the most visible and emotionally distressing side effects of breast cancer treatment. Although it is often considered temporary, growing evidence suggests that some patients experience persistent hair loss long after therapy completion. In this review, we examined published studies reporting the incidence and severity of alopecia across commonly used chemotherapy regimens for breast cancer. Our analysis shows that anthracycline- and taxane-based treatments are associated with the highest rates of severe hair loss, while other agents such as eribulin, capecitabine, vinorelbine, and gemcitabine tend to cause lower rates of alopecia. These findings highlight the importance of patient counseling, risk assessment, and survivorship care for patients undergoing breast cancer treatment.

## 1. Introduction

Breast cancer is the most commonly diagnosed malignancy in women worldwide, with a lifetime risk of approximately one in eight and more than 2 million new cases diagnosed annually [1,2,3,4]. Advances in screening, systemic therapy, and multidisciplinary care have led to substantial improvements in survival, shifting clinical attention toward long-term treatment-related toxicities and survivorship outcomes [5,6,7]. Although antibody–drug conjugates (ADCs) and other targeted therapies are increasingly incorporated into breast cancer treatment, cytotoxic chemotherapy remains a cornerstone of care in high-risk early-stage and advanced disease, and continues to account for the majority of treatment-related alopecia [5]. As survival continues to improve, the burden of chemotherapy-related adverse effects that persist beyond treatment completion has become increasingly clinically relevant.

Chemotherapy-induced alopecia (CIA) is among the most common and visible toxicities of breast cancer treatment [8]. CIA results from cytotoxic injury to rapidly dividing hair matrix keratinocytes during the anagen phase, which encompasses approximately 90% of scalp hair follicles at any given time [9]. While CIA is often perceived as a transient and unavoidable side effect of treatment, its incidence, severity, and reversibility vary substantially across agents, doses, schedules, and combination regimens [9].

Beyond its physical manifestations, CIA carries significant psychosocial and clinical consequences. Hair loss is consistently cited by patients as one of the most distressing aspects of chemotherapy, adversely affecting self-image, social functioning, and quality of life [10]. Anticipatory anxiety surrounding alopecia may influence treatment decision-making, preparedness for chemotherapy, and, in some cases, contribute to treatment delay or refusal [11,12].

While hair regrowth typically occurs following cessation of therapy, growing evidence indicates that alopecia is not universally reversible. Persistent chemotherapy-induced alopecia (pCIA), generally defined as incomplete of absent hair regrowth six months or more after completion of chemotherapy, has emerged as a clinically meaningful survivorship toxicity [10]. pCIA has been reported most frequently in breast cancer survivors, particularly following taxane- and anthracycline-containing regimens, and may present with diffuse thinning and follicular miniaturization resembling androgenetic alopecia [13,14,15]. Despite its impact, pCIA remains underrecognized, underreported, and inconsistently documented in oncology trials.

Existing data on CIA in breast cancer are fragmented across drug classes, treatment combinations, and study designs, with substantial heterogeneity in outcome definitions, grading systems, and duration of follow-up. This lack of standardized reporting hinders patient counseling, expectation-setting, and the development of targeted preventive strategies. To address this gap, we conducted a scoping review focused exclusively on breast cancer cohorts to synthesize available evidence on the incidence, severity, and persistence of CIA across commonly used chemotherapeutic regimens. This review aims to clarify regimen-specific alopecia risk and to reframe CIA, particularly pCIA, as an important and underrecognized survivorship concern in breast cancer care.

## 2. Methods

This scoping review was conducted to synthesize available evidence on the incidence, severity, and persistence of CIA in breast cancer patients across commonly used systemic treatment regimens. A scoping approach was selected to accommodate the substantial heterogeneity in study design, chemotherapy protocols, alopecia definitions, grading systems, and follow-up duration present in the existing literature.

A comprehensive literature search was performed using PubMed, EMBASE, SCOPUS, and Cochrane Library through October 2025. Search terms included combinations of keywords related to alopecia and chemotherapy, including “chemotherapy-induced alopecia,” “hair loss,” “breast cancer,” and specific chemotherapy classes and agents such as “anthracyclines,” “taxanes,” “alkylating agents,” “antimetabolites,” and “vinca alkaloids.” Boolean operators were applied to refine and combine search terms.

Studies were eligible for inclusion if they were peer-reviewed clinical trials, prospective or retrospective cohort studies, or observational analyses reporting CIA incidence, severity, or persistence in patients receiving systemic chemotherapy for breast cancer. Case reports, reviews, commentaries, and studies that did not report alopecia-related outcomes were excluded. Studies involving non-breast cancer populations or non-cytotoxic therapies without chemotherapy exposure were also excluded.

Titles and abstracts were independently screened for relevance, followed by full-text reviews of potentially eligible studies. Fifty-six studies encompassing diverse breast cancer populations, treatment settings, and chemotherapy regimens were included. Due to overlapping cohorts and multi-arm trial designs, quantitative pooling of patient numbers by individual regimen was not performed.

For each included study, data were extracted on study design, sample size, patient population, chemotherapy regimen and dosing schedule, reported CIA incidence, alopecia severity grading, and duration of follow-up when available. Alopecia severity was reported using Common Terminology Criteria for Adverse Events (CTCAE) grading when specified. Under CTCAE version 4.0 and later, alopecia is formally graded as grade 1 (<50% hair loss) or grade 2 (≥50% hair loss) [16]. However, several clinical trials and observational studies included in this review used non-standard or legacy terminology to describe alopecia severity, including references to “grade 3” or “grade 4” alopecia to denote severe or complete hair loss. In such cases, these descriptors were interpreted qualitatively as indicating extensive or near-complete alopecia, rather than as formal CTCAE grades, to avoid misclassification. Persistent CIA (pCIA) was analyzed separately as a long-term outcome and was not considered a measure of acute alopecia severity.

Results were synthesized descriptively and organized by chemotherapeutic drug class, including anthracyclines, taxanes, alkylating agents, antimetabolites, and vinca alkaloids. Regimen-specific patterns in CIA incidence, severity, and persistence were evaluated to identify clinically relevant trends (Table 1 and Table 2).

To minimize duplication and improve interpretability, alopecia outcomes were analyzed separately for monotherapy and combination regimens. Monotherapies were evaluated to characterize intrinsic alopecia risk attributable to individual agents. Combination regimens were analyzed independently, recognizing that alopecia severity in multi-agent chemotherapy reflects synergistic follicular toxicity rather than additive effects. Combination regimens were categorized based on their dominant alopecia-inducing agent.

## 3. Chemotherapy Monotherapies

### 3.1. Antimicrotubular Agents

#### 3.1.1. Taxanes: Paclitaxel and Docetaxel

Taxanes, including paclitaxel and docetaxel, were consistently associated with the highest incidence and severity of CIA among agents used in breast cancer treatment. By stabilizing microtubules and inducing prolonged mitotic arrest, taxanes disrupt proliferation in rapidly dividing cells and may additionally impair follicular stem and progenitor cell populations, contributing to both severe and persistent alopecia [17,18,19,20].

Paclitaxel monotherapy was associated with high rates of alopecia across dosing schedules. Standard three-week dosing (175–225 mg/m^2^) resulted in alopecia in 62–100% of treated patients, with severe alopecia reported in up to 87% of cases [21,22]. Weekly paclitaxel schedules were associated with lower overall alopecia incidence (approximately 30%), although alopecia remained a frequent and clinically relevant toxicity [23].

Docetaxel monotherapy was similarly associated with substantial alopecia burden. In patients with metastatic breast cancer receiving weekly docetaxel, alopecia was reported in 58% of subjects, with severe alopecia observed in 40% [24].

#### 3.1.2. Eribulin

Eribulin mesylate is a halichondrin B analog that inhibits microtubule dynamics by binding to the plus ends of microtubules and suppressing growth without stabilizing existing filaments, leading to mitotic blockade and apoptosis in rapidly proliferating cells [25,26]. This mechanism differs from taxanes and may limit collateral damage to follicular stem and progenitor cell populations [27].

In clinical studies administering eribulin (1.4 mg/m^2^ on days 1 and 8 of a 21-day cycle), alopecia was reported in 60.1% of patients and was uniformly mild-to-moderate, with no severe cases observed [28]. A phase III open-label trial including 503 women with recurrent or metastatic breast cancer reported alopecia in 44.5% of patients, consisting exclusively of grade 1 (26%) and grade 2 (17%) events [29]. An alternative dosing schedule (days 1, 8, 15 of a 28-day cycle), resulted in alopecia in 40% of patients, again limited to grade 1–2 severity [30].

### 3.2. Anthracyclines: Doxorubicin and Epirubicin

Anthracyclines, including doxorubicin and epirubicin, were associated with high rates of CIA across breast cancer cohorts. These agents exert cytotoxic effects through DNA intercalation, inhibition of topoisomerase II, and generation of reactive oxygen species (ROS), leading to apoptosis in rapidly proliferating cells such as anagen-phase hair follicle keratinocytes [31,32,33]. Experimental and clinical data further suggest that oxidative stress, mitochondrial dysfunction, follicular vascular injury, and sebaceous gland degeneration contribute to anthracycline-induced follicular damage [34,35].

Doxorubicin-induced alopecia was reported in approximately 60–100% of treated patients, with onset typically occurring within two to four weeks of treatment initiation [36]. Conventional doxorubicin administered at standard doses (60–75 mg/m^2^ every three weeks) resulted in alopecia in 35–80% of patients, with severe alopecia reported in up to 61% [37,38,39]. In contrast, pegylated liposomal doxorubicin was associated with substantially lower alopecia incidence, with some studies reporting rates as low as 20%; severe alopecia was uncommon (7%), highlighting the potential protective effect of altered pharmacokinetics on hair follicles [37].

Epirubicin demonstrated alopecia rates comparable to or exceeding doxorubicin. Across trials involving patients with locally advanced or metastatic breast cancer, alopecia was reported in 87–94% of patients, with most cases classified as moderate-to-severe [40,41]. High-dose regimens were associated with severe alopecia in more than 85% of patients [41].

### 3.3. Lower Incidence Regimens: Alkylating Agents, Antimetabolites, and Vinca Alkaloids

Alkylating agents, including cyclophosphamide, induce DNA damage by intercalating between strands and generating reactive oxygen species (ROS), contributing to tissue damage and toxicity [42]. Cyclophosphamide monotherapy in women with stage II breast cancer was associated with notable CIA incidence, with 66% of patients reported alopecia of any grade [43]. However, cyclophosphamide is more commonly administered in combination regimens, where alopecia incidence is substantially higher.

Antimetabolites, such as capecitabine, were associated with relatively low rates of CIA when administered as monotherapy. These agents disrupt nucleotide metabolism by mimicking natural pyrimidines, ultimately interfering with DNA and RNA synthesis [44,45]. Capecitabine monotherapy was associated with alopecia in approximately 8–24% of patients, with hair loss predominantly described as mild-to-moderate in severity; severe alopecia was reported in less than 1% of patients [46,47,48].

Vinca alkaloids, including vinorelbine, disrupt microtubule assembly and arrest cells in metaphase, but appear to exert less consistent cytotoxicity toward rapidly cycling hair follicle keratinocytes than taxanes. As a result, vinca alkaloids were associated with a comparatively limited alopecia burden across breast cancer cohorts. Vinorelbine monotherapy resulted in alopecia in approximately 10–23% of patients, with hair loss typically limited to mild-to-moderate severity; severe alopecia was rarely reported [49,50,51,52,53].

## 4. Combination Chemotherapy Regimens

Combination regimens were analyzed separately to account for synergistic follicular toxicity observed with multi-agent chemotherapy. In these settings, alopecia incidence reflects cumulative exposure and the dominant alopecia-inducing agent.

### 4.1. Antimicrotubular-Based Combinations

Taxane-containing combination regimens were consistently associated with high rates of acute CIA across breast cancer treatment settings. Regimens incorporating docetaxel or paclitaxel frequently produced severe and, in many cases, near-complete hair loss, irrespective of accompanying cytotoxic or biologic agents.

Alopecia was severe and universal in combinations of paclitaxel and doxorubicin (98–100%), with and without G-CSF treatment [54,55]. In locally advanced or early HER2-negative breast cancer patients, paclitaxel and epirubicin combination yielded overall alopecia in 91.9% of subjects [56]. A phase III randomized trial compared the FEC-P regimen (5-FU, epirubicin, cyclophosphamide for 3 weeks followed by paclitaxel, for four cycles) compared to EC-P (epirubicin and cyclophosphamide, followed by paclitaxel, for four cycles) both as standard chemotherapy and as dose-dense regimens, of which toxicities were highly comparable (44–48% alopecia overall) [57]. One trial randomly assigned metastatic breast cancer patients to receive paclitaxel or docetaxel combined with non-pegylated liposomal anthracycline every four weeks, resulting in complete alopecia in 83% of patients [58].

Paclitaxel combined with gemcitabine given over a three-week cycle caused severe alopecia in 75% of patients [59]. However, in a multicenter phase III trial, only 36% of patients developed alopecia, which was only grade 1–2 in severity [60]. Conversely, the same regimen given at weekly intervals drastically reduced the severity of alopecia, with 60% of reported alopecia being classified as mild-to-moderate and only 4% as severe-to-complete [61].

In patients with BRCA1/2 locally recurrent or metastatic breast cancer, patients treated with paclitaxel and carboplatin combination reported overall alopecia in 58.3% (1% severe), compared to the triple combination with veliparib which caused alopecia in 68.8% of patients (1.1% severe) [62]. Weekly paclitaxel combined with capecitabine and bevacizumab resulted in alopecia in 66% of patients [63]. Conversely, nanoformulated paclitaxel (Nab-paclitaxel) combined with trastuzumab and pertuzumab still yielded significant alopecia, affecting 55.6% of HER2-positive metastatic breast cancer patients [64].

Similarly, docetaxel and capecitabine combination caused alopecia in 54.2% [65]. Docetaxel combined with cyclophosphamide produced overall alopecia in 76.3% of patients [66]. Docetaxel combined with gemcitabine resulted in alopecia in 34.5% of metastatic breast cancer patients when administered weekly, with severe alopecia observed in 8.6% [67]. In contrast, the same combination administered at biweekly intervals resulted in alopecia in 72% of patients, including complete hair loss in 35% [68]. A phase II study using a similar biweekly schedule reported grade 1–2 alopecia in 78% of patients [69].

Treatment of advanced breast cancer with docetaxel and vinorelbine with or without G-CSF, yielding overall alopecia in 76% of patients, with 32% being severe [70]. In two trials, docetaxel was combined with trastuzumab and pertuzumab in HER2-positive metastatic breast cancer, in which overall alopecia ranged from 46.8 to 53.8% of patients [64,71].

Eribulin combined with cyclophosphamide resulted in mild-to-moderate alopecia in 31.8% of advanced breast cancer patients in a phase Ib/II study [72]. A phase II study combined eribulin with gemcitabine for the treated of locally advanced or metastatic triple negative breast cancer, in which 23.8% of patients developed alopecia, with only 3.6% classified as severe [73]. Conversely, the triple combination of eribulin with doxorubicin and cyclophosphamide as neoadjuvant therapy for patients with HER2-negative inflammatory breast cancer caused alopecia in 63.6% of patients, of which 54.5% was categorized as grade 2 severity [74].

In a multicenter phase II trial, eribulin combined with bevacizumab in patients with HER2-negative metastatic breast cancer resulted in alopecia in 16% of patients, with 13% classified as mild-to-moderate and 3% as severe [75]. Similarly, the combination of eribulin with nivolumab in HER2-negative metastatic breast cancer caused grade 1–2 alopecia in 13.3% of patients [76]. However, a phase Ib/II trial in triple-negative breast cancer patients treatment with eribulin and pembrolizumab resulted in grade 1–2 alopecia in 39.5% of subjects [77].

### 4.2. Anthracycline-Based Combinations

Combination regimens including anthracyclines without taxanes still produced significant alopecia. Doxorubicin and cyclophosphamide regimens resulted in nearly universal alopecia (92.4%), with 69.5% being severe [78]. Dose-dense doxorubicin and cyclophosphamide chemotherapy combined with pegteograstim yielded lower alopecia incidence, with only mild to moderate alopecia reported in 43% of patients [79]. Comparatively, a randomized multicenter phase II trial assessed the efficacy of non-pegylated liposomal doxorubicin combined with cyclophosphamide versus a metronomic schedule of both in untreated HER2-negative advanced breast cancer [80]. The standard treatment caused alopecia in 77% of patients compared to the metronomic regimen, which caused significantly less alopecia (27%); notably, no patients reported severe alopecia in either regimens.

Triple combination of doxorubicin, cyclophosphamide, and 5-FU (FAC) was assessed in metastatic breast cancer patients, of which 93.4% had near-universal alopecia; however, only 22.2% was severe [81]. Similar results were observed in a study of adjuvant chemotherapy for operable breast cancer, in which 93.5% of patients treated with the same FAC regimen had overall alopecia, with 69.5% being severe [82].

The combination of epirubicin and cyclophosphamide in HR-positive, HER2-negative breast cancer resulted in alopecia in 90% of patients [66]. Weekly doxorubicin and continuous infusional 5-FU caused alopecia in all advanced breast cancer patients, of which 98% was severe [83]. High-dose epirubicin (20 mg/m^2^) and gemcitabine combination yielded severe alopecia in 40% of patients, while severe alopecia was not reported with lower dosages of epirubicin (10–15 mg/m^2^) [84]. These findings were corroborated by a phase II study in patients with locally advanced or metastatic breast cancer treated with epirubicin (15 mg/m^2^) and gemcitabine (1000 mg/m^2^) administered weekly for three weeks followed by one week rest, in which 38.2% of patients developed severe alopecia [85].

Triple combination of epirubicin with cyclophosphamide and 5-FU was assessed in a metastatic breast cancer cohort to compare a monthly versus a weekly regimen [86]. Alopecia was reported in 93% of patients in the monthly treatment arm compared to 71% in the weekly group. Notably, severe alopecia was significantly more prevalent and earlier onset in the monthly group (71%) compared with the weekly treated group (14%; *p* < 0.0001). Another clinical trial assessed the same triple combination chemotherapy as a monthly regimen in advanced breast cancer cohort, reporting severe alopecia in 86% of patients [87].

### 4.3. Lower Alopecia Risk Combinations

Cyclophosphamide-containing regimens, without taxanes or anthracyclines, yielded moderate overall alopecia incidence, generally with lower severity. In cyclophosphamide, methotrexate and 5-FU combination regimens (CMF) resulted in overall alopecia in 71.4% of patients, but only 15.1% was severe [78]. However, two other studies assessing the same regimen reported alopecia ranging from 36 to 43% of patients, with only 2% being severe [43,82].

A dose-escalation study evaluated the combination of vinorelbine and capecitabine in women with pre-treated advanced-stage breast cancer [88]. Lower dosages (vinorelbine: 12.5–20 mg/m^2^, capecitabine: 500–1125 mg/m^2^) caused mild-to-moderate alopecia in 30.8% of patients compared to 40% in the higher dosage arm (vinorelbine: 20–25 mg/m^2^, capecitabine: 1250 mg/m^2^), with no severe alopecia reported in either group. Conversely, vinorelbine combined or alternating with capecitabine resulted in alopecia in 18.2% of those in the combined arm and 13.0% in the alternating arm [65]. In another study, vinorelbine and gemcitabine in combination resulted in grade 2 alopecia in only one patient (1.88%), though grade 1 hair loss incidence was not collected [89]. Moreover, the combination of cisplatin and gemcitabine, as first-line therapy for metastatic triple-negative breast cancer, resulted in mild-to-moderate alopecia in 10% of patients [60].

As for triple combinations consisting of 5-FU, vinorelbine, and cisplatin, mild-to-moderate alopecia was observed in 24% of patients, and no severe cases were observed [90]. When substituting cisplatin for folinic acid, alopecia was reported in 35% of patients, though both 5-FU and vinorelbine were administered at higher dosages [91]. In a phase II trial of gemcitabine and carboplatin combination with trastuzumab in HER2-positive metastatic breast cancer patients, alopecia was infrequent, with only 34% developing grade 1 hair loss and 3% grade 2 [92].

## 5. Persistent Alopecia Risk

### 5.1. Taxanes

Persistent chemotherapy-induced alopecia (pCIA) following taxane treatment is an increasingly recognized complication. A retrospective survey at two tertiary UK cancer centers reported 10.1% of paclitaxel-treated patients and 23.3% of docetaxel-treated subjects developed significant pCIA between one and seven years of treatment completion [93]. Interestingly, non-scalp hair involvement was more frequently described in paclitaxel-treated patients (4.3%) than in docetaxel-treated patients (1.8%, *p* = 0.29). However, more recent data demonstrates higher prevalence of pCIA, especially in docetaxel and cyclophosphamide combinations or TCHP regimens, with rates reaching up to 52% [94]. Similarly, a prospective study identified the FEC and docetaxel combination as also causing pCIA in 20 Caucasian females [95]. Another study evaluated pCIA severity across regimens and found that docetaxel at cumulative doses ≥400 mmg/m^2^ was associated with grade 1 pCIA in 33–52% and grade 2 pCIA in 5–12% of patients [15]. Sequential anthracycline-docetaxel regimens, including epirubicin and cyclophosphamide followed by docetaxel (EC-T), produced pCIA in 30% of patients, compared to 14% in those treated with the combination of epirubicin and docetaxel followed by capecitabine (ET-X) [96].

### 5.2. Anthracyclines

Although most data involving anthracyclines and pCIA is typically associated with combination regimens with taxanes, few studies have assessed the incidence of pCIA isolated to anthracyclines. Doxorubicin-containing regimens (i.e., AC) were associated with high rates of pCIA, reported in up to 67% of treated patients [94]. However, in another cohort involving 100 patients with pCIA, only five had undergone epirubicin-based treatment in the absence of taxanes [97]. Therefore, the isolated contribution of anthracyclines to pCIA risk remains uncertain.

## 6. Discussion

CIA remains one of the most visible and psychologically impactful toxicities of breast cancer treatment, yet its clinical significance has historically been underestimated. Although alopecia is often regarded as a transient and unavoidable consequence of cytotoxic therapy, our synthesis of regimen-specific data demonstrates that both the severity and persistence of hair loss vary substantially by drug class, dose intensity, and combination strategy. Importantly, these findings challenge the long-standing assumption that CIA is uniformly reversible and position pCIA as a clinically meaningful survivorship toxicity.

Across the available literature, anthracycline- and taxane-containing regimens were associated with the highest rates of acute CIA, with severe alopecia frequently approaching 90–100% in dose-dense or multi-agent protocols [21,22,36,40,41]. Alkylating agents consistently amplified alopecia severity when administered in combination with anthracycline or taxanes, contributing to the near-universal hair loss in several clinical trials [66,78,98]. In contrast, antimetabolites (i.e., capecitabine) and vinca alkaloids (i.e., vinorelbine) were associated with substantially lower alopecia incidence and severity, particularly when administered outside of taxane-containing combinations [65,88]. More recently incorporated cytotoxic agents used in contemporary breast oncology, including eribulin and gemcitabine, similarly demonstrated lower severity alopecia profiles. Alopecia reported with eribulin monotherapy was predominantly mild-to-moderate, while hair loss observed in gemcitabine-containing regimens appeared largely attributable to concomitant agents rather than gemcitabine itself [28,29,30,60,73,85,92]. These findings emphasize that CIA risk is not solely agent-specific but is strongly influenced by cumulative exposure and synergistic toxicity within multi-drug regimens.

Anthracyclines and taxanes exhibit biological properties that predispose to both severe acute alopecia and long-term follicular injury. These agents induce apoptosis in rapidly proliferating cells, leading to extensive damage of anagen hair matrix keratinocytes, thereby causing rapid apoptosis and follicular dystrophy [9]. However, accumulating evidence suggests that irreversible alopecia arises when cytotoxic damage extends beyond the matrix to involve follicular stem and progenitor cell populations that are essential for hair regeneration [97,99]. Taxanes, particularly docetaxel, induce sustained microtubule stabilization and prolonged mitotic arrest, effects that may impair stem cell viability and disrupt normal follicular cycling [17,18,19,20]. In contrast, eribulin’s suppression of microtubule growth without prolonged stabilization may limit collateral injury to follicular stem and progenitor cells, offering a biologically plausible explanation for the consistently lower severity of alopecia observed across eribulin-based regimens. Anthracyclines, through DNA intercalation and oxidative stress, may similarly produce cumulative follicular injury that exceeds the regenerative capacity of the hair follicle, particularly in dose-dense or combination regimens [31,32,33].

In addition to direct cellular toxicity, high incidence agents may disrupt the follicular microenvironment. Injury to perifollicular vasculature, sebaceous glands, and signaling networks that regulate stem cell quiescence and activation has been implicated in impaired hair regrowth [100,101]. These changes may promote prolonged telogen arrest and follicular miniaturization, clinical features commonly observed in pCIA. The likelihood of irreversible injury appears further influenced by cumulative exposure, high-dose intensity, and sequential administration of anthracyclines followed by taxanes, consistent with the elevated rates of pCIA reported in these settings. Together, these mechanisms provide a biologically plausible explanation for the disproportionately high rates of both severe acute CIA and pCIA observed with anthracycline- and taxane-containing regimens.

In contrast, agents associated with lower alopecia incidence demonstrate pharmacologic properties that may limit irreversible follicular injury. Drugs such as capecitabine, an oral prodrug of 5-FU that inhibits thymidylate synthase during DNA synthesis, and vinorelbine, a vinca alkaloid that inhibits microtubule polymerization with lower mitotic arrest potency compared with taxanes, demonstrate greater cell-cycle specificity and reduced toxicity toward quiescent or slowly cycling cell populations, including follicular stem and progenitor cells [88,102,103]. As a result, cytotoxic effects may be largely confined to the hair matrix, allowing for subsequent follicular recovery once treatment is discontinued. This distinction supports the concept that reversible alopecia reflects transient matrix injury, whereas persistent alopecia arises when regenerative compartments of the follicle are compromised.

These findings support the concept that pCIA represents a threshold phenomenon rather than an extension of acute alopecia severity. When cytotoxic injury remains confined to the matrix, regrowth is typically observed. However, once regenerative follicular compartments are compromised, recovery may be incomplete or absent. This distinction helps explain why only a subset of patients exposed to high-risk regimens develop pCIA and underscores the importance of cumulative dose intensity and sequential combination therapy in determining long-term follicular outcomes. Moreover, emerging data indicate that anthracyclines should not be considered benign with respect to long-term alopecia [94]. Although taxanes have been most consistently implicated in pCIA, emerging evidence shows that doxorubicin-containing regimens may confer similar risks of persistent alopecia. These observations challenge the prevailing view that anthracycline-associated alopecia is uniformly reversible and suggest that cumulative follicular injury from anthracyclines, particularly in dose-dense or combination settings, may result in lasting hair loss.

Within this mechanistic framework, the evolving role of ADCs in breast cancer therapy warrants consideration. Although ADCs are often regarded as less toxic than conventional chemotherapy because they concentrate the drug at antigen-expressing tumor cells, they deliver highly potent cytotoxic payloads capable of affecting rapidly dividing non-malignant tissues, including hair follicle keratinocytes [104]. As such, ADCs such as trastuzumab deruxtecan and trastuzumab emtansine have the potential to affect hair follicle keratinocytes through mechanisms that overlap with those of traditional cytotoxic chemotherapy, including microtubule disruption and DNA damage [105]. Available clinical data suggest that alopecia associated with ADCs is generally less frequent and less severe than that observed with taxane- or anthracycline-based regimens; however, alopecia outcomes remain inconsistently reported and severity is rarely characterized [105,106,107]. Importantly, alopecia has not been meaningfully observed with trastuzumab or pertuzumab when administered in the absence of chemotherapy, underscoring the central role of cytotoxic injury, rather than HER2-targeted signaling inhibition, in treatment-related hair loss [108]. As the use of ADCs expands across both early-stage and metastatic breast cancer, systematic reporting of alopecia incidence, severity, and persistence will be essential to accurately define survivorship risk and to prevent under-recognition of long-term toxicities analogous to pCIA observed with conventional chemotherapy.

Moreover, the psychosocial burden of CIA extends far beyond cosmetic concern. Hair loss is consistently cited by patients as one of the most distressing aspects of chemotherapy, with significant adverse effects on self-image, social functioning, and overall quality of life. In breast cancer survivors with pCIA, Dermatology Life Quality Index (DLQI) scores are substantially elevated compared with the general population (mean 8.66 vs. 0.5), with nearly 40% of patients reporting severe impairment in quality of life [95]. This degree of psychosocial morbidity underscores that alopecia represents a clinically meaningful survivorship challenge rather than a minor or transient adverse event.

The impact of CIA is particularly relevant to breast cancer, where the vast majority of affected individuals are women [109]. Hair is closely tied to perceptions of femininity, youth, and sexuality, and its loss has been associated with feelings of shame, reduced self-confidence, and altered self-identity [11,110,111,112]. Because hair loss functions as a visible marker of illness, persistent alopecia may serve as a chronic reminder of prior cancer treatment, reinforcing its role as a long-term survivorship concern rather than a temporary toxicity. This visible and enduring nature of pCIA may amplify its psychological burden compared with other chemotherapy-related toxicities that resolve following treatment completion.

Despite its prevalence and impact, CIA remains inadequately characterized in clinical trials and survivorship care. Alopecia is frequently reported as a secondary or non-prioritized adverse event, with heterogeneous grading systems and limited long-term follow-up. This lack of standardized reporting has contributed to the underrecognition of pCIA and limits accurate counseling regarding regimen-specific risk. Furthermore, preventive strategies remain limited. Scalp cooling has demonstrated efficacy in reducing acute CIA in taxane-based regimens, but access, cost, and variable effectiveness restrict its widespread adoption [113,114]. Pharmacologic approaches remain investigational, and no therapies are currently approved for the prevention or treatment CIA and pCIA. Given these limitations, knowledge of regimen-specific CIA risk is essential for counseling, expectation-setting, and survivorship planning.

This review has several limitations. As a scoping review, findings were synthesized descriptively without quantitative pooling, precluding formal meta-analytic risk estimates. Substantial heterogeneity exists across included studies with respect to chemotherapy regimens, dosing schedules, patient populations, follow-up duration, and alopecia assessment methods. Importantly, alopecia grading was inconsistently reported, with some studies using formal CTCAE v4.0 criteria, whereas others relied on legacy or non-standard terminology (e.g., “grade 3” or “grade 4” alopecia) despite current CTCAE classification limiting alopecia to grade 1 or grade 2. These inconsistencies introduce potential misclassification and limit cross-study comparability. Additionally, overlapping cohorts and multi-arm trial designs preclude precise attribution of alopecia incidence to individual agents within combination regimens. Long-term follow-up for pCIA was inconsistently reported, likely resulting in underestimation of survivorship burden.

Future research should prioritize prospective studies with standardized alopecia endpoints, regimen-stratified analyses, and extended follow-up to better define risk factors for both acute and persistent hair loss. Integration of patient-reported outcomes will be essential to capture the functional and psychosocial dimensions of CIA. Mechanistic studies focused on follicular stem cell injury, cumulative cytotoxic exposure, and taxane-specific toxicity may further elucidate pathways underlying irreversible alopecia and inform targeted preventive strategies. Recognizing CIA, particularly pCIA, as a meaningful survivorship toxicity is critical to improving patient counseling, expectation-setting, and quality of life for the growing population of breast cancer survivors.

## 7. Conclusions

CIA is among the most frequent toxicities of breast cancer treatment, with incidence often exceeding 80–90% in patients receiving anthracycline- and taxane-based regimens. Cyclophosphamide further amplifies acute alopecia when combined with these agents, contributing to the near-complete hair loss observed in AC- and TC-based protocols. Although traditionally regarded as temporary, emerging evidence demonstrates that pCIA may affect a substantial proportion of survivors, including up to 67% of patients treated with doxorubicin-containing regimens and nearly half of those receiving docetaxel-based combinations. In contrast, capecitabine- and vinorelbine-based therapies confer comparatively lower risk of both acute and long-term alopecia.

These regimen-specific patterns establish CIA not only as a visible treatment toxicity but, in some cases, as a clinically meaningful survivorship complication with lasting implications for quality of life and psychosocial well-being. Despite its prevalence, CIA remains inconsistently reported in oncology trials and lacks standardized preventive strategies. Improved surveillance, standardized reporting, and the development of targeted preventive interventions are urgently needed to reduce the burden of this highly prevalent yet underrecognized toxicity in breast cancer care.

## Figures and Tables

**Table 1:** Alopecia risk with chemotherapy monotherapy in breast cancer. CIA incidence rated as low (<30%), moderate (30–59%), high (60–89%), near-universal (≥90%). Severity rated as mild-moderate, moderate-severe, or severe/near-complete.

Drug Class	Agent	CIA Incidence	Acute Alopecia Severity
Antimicrotubular Agents	Paclitaxel	Near-Universal	Severe to Near-Complete
	Docetaxel	Moderate	Moderate to Severe
	Eribulin	Moderate	Mild to Moderate
Anthracyclines	Epirubicin	Near-Universal	Severe to Near-Complete
	Doxorubicin	High	Moderate to Severe
Alkylating Agents	Cyclophosphamide	High	Mild to Severe
Antimetabolites	Capecitabine	Low	Mild to Moderate
Vinca Alkaloids	Vinorelbine	Low	Mild to Moderate

**Table 2:** Combination chemotherapy regimens associated with moderate to high incidence and/or clinically significant alopecia in breast cancer. CIA incidence rated as low (<30%), moderate (30–59%), high (60–89%), and near-universal (≥90%). Severity rated as mild-moderate, moderate-severe, or severe/near-complete. Combination regimens were categorized based on the dominant alopecia-inducing agent.

Dominant Agent	Regimen	CIA Incidence	Acute Alopecia Severity	pCIA
Antimicrotubular Agents	Paclitaxel + Doxorubicin	Near-Universal	Severe to Near-Complete	10–52%
	Docetaxel + Cyclophosphamide	High	Severe	
	Epirubicin + Cyclophosphamide → Paclitaxel	Moderate	Moderate to Severe	
	Docetaxel + Carboplatin + Trastuzumab + Pertuzumab	Moderate	Moderate to Severe	
	Paclitaxel + Gemcitabine	High	Moderate to Severe	N/A
	Docetaxel + Gemcitabine	High	Moderate to Severe	
	Eribulin + Doxorubicin + Cyclophosphamide	High	Moderate	
Anthracycline	Epirubicin + 5-FU	Near-Universal	Severe to Near-Complete	5–67%
	Epirubicin + Cyclophosphamide + 5-FU	Near-Universal	Severe to Near-Complete	
	Doxorubicin + Cyclophosphamide	High	Severe	
	Doxorubicin + Cyclophosphamide + 5-FU	High	Severe	

## Data Availability

No new data were created or analyzed in this study. Data sharing is not applicable to this article.
